# Barriers, facilitators and solutions for active inclusive play for children with a physical disability in the Netherlands: a qualitative study

**DOI:** 10.1186/s12887-021-02827-5

**Published:** 2021-08-28

**Authors:** L. van Engelen, M. Ebbers, M. Boonzaaijer, E. A. M. Bolster, E. A. H. van der Put, M. A. T. Bloemen

**Affiliations:** 1 HU University of Applied Sciences Utrecht, Institute of Human Movement Studies, Master Pediatric Physiotherapy, Utrecht, The Netherlands; 2 HU University of Applied Sciences Utrecht, Research Group Lifestyle and Health, Research Centre Healthy and Sustainable Living, Utrecht, The Netherlands; 3 De Speeltuinbende, Amsterdam, The Netherlands

**Keywords:** Children with physical disabilities, Inclusive active play, Pediatric physical therapy

## Abstract

**Background:**

Children with physical disabilities (PD) are less physically active than typically developing peers. The most important contributor to physical activity for primary school-aged children is outside play and therefore this should be part of every child’s life. However, children with PD experience multiple barriers to participation in playgrounds. Despite recent improvements in the accessibility of Dutch playgrounds, the participation of children with PD has not increased. This study aims to explore facilitators, barriers and solutions influencing the participation of children with PD in Dutch outdoor playgrounds, from parents’ and professionals’ perspectives.

**Methods:**

Twelve semi-structured interviews with parents of children with PD aged 2–12 years and five focus group meetings with professionals working with these children were conducted. To ensure data saturation, we performed three member-check meetings. Two independent researchers analyzed the data using an inductive thematic approach.

**Results:**

Similar barriers, facilitators and solutions were mentioned by parents and professionals. Three main themes were identified: the emotional barrier versus the physical barrier, play as a part of an inclusive society and the role of professionals in facilitating active inclusive play. The most important personal factors were physical and social problems experienced when children with PD wanted to join outdoor play. Interestingly, parents and professionals believed the social barrier was far more important than the physical one. The most important environmental factor was that the Dutch society is not sufficiently inclusive.

**Conclusions:**

According to both parents and professionals, the most important barrier to active inclusive outdoor play was social, hindering the participation of children with PD in play with typically developing peers. To overcome such problems, professionals should take an active role in empowering children with PD and their parents. Furthermore, it is important to introduce outdoor active play early, so it becomes part of normal daily life. In addition, a change in the mindset of typically developing children and their parents seems essential to achieve true inclusive active play.

**Supplementary Information:**

The online version contains supplementary material available at 10.1186/s12887-021-02827-5.

## Background

Between 109,000 and 129,000 children in the Netherlands have a disability, roughly 3.5% of children aged 0–18 years [1]. In the last decade, the importance of play for children with physical disabilities (PD) has become more and more recognised. Play is so important it is included in the United Nations Convention on the Rights of the Child: *“The Committee … emphasizes the obligations of States parties to ensure that children with disabilities have equal access with other children to participation in play … Pro-active measures are needed to remove barriers and promote accessibility to and availability of inclusive opportunities for children with disabilities to participate in all these activities”* [2]. Although this quote implies that children with disabilities are entitled to opportunities to play, these opportunities are not always available.

When playing, children develop motor skills, learn to take risks, push their limits, interact with peers, and learn social norms and values [3, 4]. Neighborhood and school playgrounds are important sites where children play and very suitable places to be physically active [5–8]. Play in the playground is the most important contributor to physical activity for primary school-aged children [9]. A physically active lifestyle is beneficial, especially for people with disabilities [10–14]. Being physically active, including physically active play, has positive effects on motor, social and cognitive development, and on overall health [15–17]. A physically active lifestyle leads to reduced risks of illness and mortality from various chronic illnesses [15–17]. In particular, physical activity has positive effects on the functional independence of children with disabilities, improving their integration into society and their quality of life [11]. Nowadays, typically developing children lack physical activity [18–20] and children with disabilities are even less physically active than their peers [19–22].

Although playgrounds have the potential to contribute to physical activity for children with disabilities, playgrounds, in general, are insufficiently accessible for children with PD [23–28]. Physical inaccessibility is mainly caused by inappropriate ground surfaces (e.g. grass, sand, or uneven) and inaccessible play equipment, leading to reduced participation in play of children with PD, compared to their typically developing peers [23–28].

‘The Playground Gang’ (De Speeltuinbende), an initiative of the Dutch Foundation for the Handicapped Child (NSGK), strives to create accessible playgrounds for children with PD. Since the main focus in literature [23–28] is that children with PD do not participate in the playground because of physical inaccessibility, you expect increased participation of children with PD when accessibility is improved. However, even when ‘The Playground Gang’ improved the physical accessibility of playgrounds, the number of children with PD in playgrounds did not increase. Thus, other factors than physical inaccessibility may influence participation of children with PD in outdoor play negatively. Research by the LUDI network (international research consortium to increase participation in play for children with disabilities) found, even when play locations are physically accessible, they can still be socially exclusive due to social and attitudinal barriers [28]. In each country there are differences in cultural and societal structures, influencing social and attitudinal barriers. In the Netherlands for example, many children with PD attend special schools (mostly further away from home) instead of regular schools located in the neighborhood. To understand possible solutions and to create interventions to stimulate inclusive outdoor play in the Netherlands, research specific to the Dutch situation is needed.

The focus of this study will be on parents of children with PD and professionals. According to the Family-Centered Care approach, parents are the heart of the family and have by far the most important role in stimulating their child to play outside [29]. The perspective of professionals is also valuable since they work closely with children with PD and their parents. In addition, they have a role in facilitating inclusive play in the playground [30–32]. Insight into their perspectives is necessary to develop effective intervention strategies to improve participation in the playground.

The objective of this study is to analyze facilitators, barriers and solutions important for active and inclusive outdoor play for children with PD (aged 2–12 years), from both the parental and professional perspectives.

## Methods

### Design and data collection

This case study employed a qualitative descriptive design, with a thematic analysis [33] and has a basis in social constructivism [34, 35]. In this approach, questions remain broad to encourage participants to construct their meaning, supported by the interaction with others (the social part of social constructivism) [34, 35]. The Consolidated criteria for reporting qualitative studies (COREQ) was used to describe the method of this study (Additional file 1) [36]. The focus of this study is on parental and professional perspectives on participation in physically active play of children with PD aged 2–12 years with a physical disability comparable to a score of I-IV on the Gross Motor Function Classification System (GMFCS) for children with cerebral palsy [37]. How play and playgrounds were defined in this study was explained to the participants at the start of the interview (included in the topic lists, for more information see Additional file 2 and Additional file 3). The parents and professionals were interviewed separately with comparable topic lists, so any differences and similarities between these groups could become evident. However, during data collection, the topic lists were adjusted due to usability reasons, and gained knowledge related to the aim of this study. The topic lists were created by the research team, consisting of one experienced (> 15 years) MSc. pediatric physical therapist/pedagogue (MBO), two experienced (> 10 and 15 years respectively) Ph.D. pediatric physical therapists (EB, MB), and two junior researchers (< 5 years) with a MSc. pediatric physical therapy background (ME and LVE). The research team was trained to conduct qualitative research and had multiple years of experience with qualitative research.

We conducted semi-structured individual interviews to obtain data from parents of children with PD. Such interviews provide participants with an opportunity to introduce their own subjects and insights [35, 38]. For parents, we chose individual interviews over focus group interviews because of practical reasons. The interviews were solely led by the moderator ME (MSc. PPT) who made field notes to make sure all topics were discussed and to register peculiarities and non-verbal reactions.

The professionals were interviewed during focus group meetings. These are semi-structured discussions to gain insight into the perceptions of participants. Focus groups were chosen since interactions in groups can help to reveal more in-depth information [35, 38]. The ideal number of participants is reported to be six to ten [39]. Focus group meetings were led by one moderator LvE (MSc. PPT). There was one observer who made fieldnotes and supported the moderator in making sure all topics of the topic list were discussed.

In all interviews, the moderator posed open questions to which the participants responded. The participants were not directed towards any particular preconceived response. The interviews were filmed and audio-taped. Both the individual interviews and the focus group interviews were conducted at various locations such as a rehabilitation center, the HU University of Applied Sciences Utrecht, a pediatric physical therapy practice, and at the participant’s home.

Data collection was continued until data saturation was achieved, meaning that no new information was collected during a subsequent interview or meeting. To assess the likelihood of saturation [39, 40], three member checks with new participants were performed after the data collection was finished. One member check group consisted of parents, the other two of professionals. When no new information arose during the member check meetings, saturation was assumed.

The Institutional Medical Ethical Screening Committee (Department of Health, HU University of Applied Sciences Utrecht) approved all methods and concluded that the present study did not fall under the Medical Research Involving Human Subjects Act, since no intervention was conducted and the burden for participants was low. All protocols were carried out according to the Good Clinical Practice Statement. All participants were provided with a standardized information letter. Prior to the individual interviews, the focus group meetings, and the member check group meetings, every participant gave written informed consent.

### Participants and procedure

We included parents of children with PD comparable to a score of I-IV on the GMFCS and aged 2–12 years old and professionals who were experienced in working with children with PD in the field of physical activity [37]. Exclusion criteria was insufficiency in the Dutch language. We used purposive sampling to assure variation in GMFCS score, and age of the children, family background and family composition. For the professionals, we included participants with different levels of experience and different professions [40]. Maximum variation of demographic origin was aimed for. Participants were recruited by flyers through different routes, including our network, treatment practices found on the internet, and by networking at a wheelchair event. To reduce the burden of travel and time, the focus groups were formed by convenience, rather than variation by age, gender, profession, or level of experience. Prior to individual interviews and focus group meetings, every participant completed a standardized questionnaire with background information (Additional file 4 and Additional file 5). Parents provided information about their children and families and professionals about their working experience.

### Data analysis

All individual interviews and focus group meetings were transcribed verbatim based on the audio- and videotapes. A thematic analysis was performed using an inductive strategy [33] with MAXQDA2018 (version 18.0.4). It was an iterative process in which fragments were coded, resulting in themes. The first step was familiarization with the data by reading the transcripts several times. The second step was identifying fragments and coding these fragments to distinguish between barriers, facilitators and solutions. Barriers and facilitators were defined as aspects already present and which have a negative or positive influence on physically active participation by children with PD in playgrounds. Solutions were defined as factors, not yet present but with a potentially positive influence on participation in playgrounds. During the third step, we searched for a broader level of themes within the codes. The fourth step specified the detailed reviewing of the themes in relation to the individual codes and to the entire data set to conclude if the themes display all the collected information. The fifth step was defining and naming the themes and distinguish themes from subthemes.

The analyses were conducted independently by two trained researchers (ME and LvE) and then compared. In the case of disagreement, a third researcher (MB) was consulted to achieve consensus. To prevent research bias, critical peer debriefing occurred after each step, and results were discussed thoroughly within the research team [39, 40].

## Results

From November 2017 to February 2018, 12 semi-structured interviews with parents and five focus group meetings with professionals (*n* = 25) were conducted. The characteristics of the parents and professionals are displayed in Table 1 and Table 2. The average duration of the interviews with parents was 46 min and 90 min for the focus group meetings. After the eleventh interview and the fourth focus group meeting, no new information was revealed. In addition, no new information was discussed by the participants (parents *n* = 5; professionals *n* = 15) during the member checks. Therefore, the researchers concluded that saturation had been achieved. No additional information emerged from the non-verbal communication (video) and the field notes, so they have not been further analyzed.

**Table 1:** Characteristics of the parents

	Age of parent (years)	Gender of parent Male (M) /Female (F)	Age child (years)	Gender of child Male (M) /Female (F)	Number of siblings	Diagnose of child	Main way of daily locomotion of the child	Degree of Parental education	Education show of child
Parent 1	35	F	5	F	1	Cerebral palsy	Walking	High	Regular
Parent 2	34	F	11	F	1	Spina bifida	Wheelchair dependent	Middle	Regular
Parent 3	38	F	5	M	0	Congenital disorder	Wheelchair dependent	High	Regular
Parent 4	38	F	11	F	0	Metabolic disease	Walking	High	Special
Parent 5	31	F	2	F	0	Developmental disorder	Walking	High	Pre school show daycare
Parent 6	31	F	5	M	2	Spina bifida	Wheelchair dependent	Middle	Regular
Parent 7	45	F	12	M	0	Spina bifida	Wheelchair dependent	High	Special
Parent 8	26	F	4	M	1	Spina bifida	Wheelchair dependent	Middle	Special
Parent 9	44	F	12	M	1	Metabolic disease	Walking	High	Special
Parent 10	30	F	6	F	2	Systematic mastocytosis	Walking	Middle	Home school
Parent 11	44	F	7	M	0	Developmental coördination disorder	Walking	Middle	Regular
Parent 12	46	F	10	F	3	Dendy walker syndrome	Walking	High	Special
Membercheck									
Parent 13	48	M	8	F	1	Cerebral palsy	Walking	High	Special
Parent 14	38	F	10	M	1	Asperger/ASS	Walking	High	Regular
Parent 15	39	F	7	M	0	Cerebral palsy	Walking	High	Special
Parent 16	Unknown	F	8	M	0	Cerebral palsy	Walking	Middle	Regular
Parent 17	46	F	11	M	3	Apert syndrome	Walking	High	Special

**Table 2:** Characteristics of the professionals

	Male(M) /Female(F)	Profession	Work setting	Work experience (years)		
Focusgroup 1						
Professional 1	M	Wheelchair skills trainer	Other	13		
Professional 2	M	Wheelchair skills trainer, Organizer of inclusive sports	Other	40		
Professional 3	F	Pediatric physiotherapist	Rehabilitation	13		
Professional 4	F	Pediatric physiotherapist	Rehabilitation	11		
			Median (Years)	13	Range (years)	11–40
Focusgroup 2						
Professional 5	F	Pediatric physiotherapist	Private practice	20		
Professional 6	M	Organizer of inclusive sports	Other	4		
Professional 7	F	Organizer of inclusive sports, Care sport connector, Gymnastic teacher	Special needs education	8		
Professional 8	F	Occupational therapist	Private practice	2		
Professional 9	M	Pediatric physiotherapist	Private practice	35		
			Median (Years)	8	Range (years)	2–35
Focusgroup 3						
Professional 10	F	Physical education teacher	Rehabilitation	2		
Professional 11	F	Occupational therapist	Rehabilitation	15		
Professional 12	F	Occupational therapist	Rehabilitation	30		
Professional 13	F	Pediatric physiotherapist	Rehabilitation	15		
Professional 14	F	Pediatric physiotherapist	Rehabilitation	25		
Professional 15	M	Gymnastic teacher	Special needs education	19		
			Median (Years)	17	Range (years)	2–30
Focusgroup 4						
Professional 16	F	Pediatric physiotherapist	Rehabilitation	4		
Professional 17	F	Pediatric physiotherapist, Personal care assistant	Private practice	2		
Professional 18	M	Pediatric physiotherapist	Rehabilitation	21		
			Median (Years)	4	Range (years)	2–21
Focusgroup 5						
Professional 19	F	Pediatric physiotherapist	Rehabilitation	37		
Professional 20	F	Pediatric physiotherapist	Private practice	5		
Professional 21	F	Occupational therapist	Private practice	1		
Professional 22	F	Occupational therapist	Private practice	1		
Professional 23	F	Pediatric physiotherapist	Private practice	3		
Professional 24	F	Gymnastic teacher	Special needs education	28		
Professional 25	F	Pediatric physiotherapist	Private practice	7		
			Median (Years)	5	Range (years)	1–37
Mebercheck 1						
Professional 26	F	Pediatric remedial therapist	Regular education	5		
Professional 27	F	Occupational therapist	Rehabilitation	8		
Professional 28	F	Pediatric physiotherapist	Rehabilitation	25		
Professional 29	F	Pediatric remedial therapist	Other	3		
Professional 30	F	Pediatric remedial therapist	Special needs education	11		
Professional 31	F	Teacher	Regular education	1		
Professional 32	M	Pediatric physiotherapist	Private practice	10		
Professional 33	F	Occupational therapist	Private practice	7		
Professional 34	M	Pediatric physiotherapist	Private practice	15		
			Median (Years)	8	Range (years)	5–25
Membercheck 2						
Professional 35	F	Pediatric physiotherapist	Rehabilitation	5		
Professional 36	F	Policy-maker	Other	29		
Professional 37	F	Societal project consultant	Other	17		
Professional 38	M	Societal project consultant	Other	12		
Professional 39	F	Employee of playground foundation	Other	10		
Professional 40	F	Employee of playground foundation	Other	15		
			Median (Years)	13,5	Range (years)	5–29

The overall results are displayed in Tables 3, 4, and 5. The most important themes are discussed in the text and supported with relevant quotes. The main themes are presented as follows:

1. The emotional barrier versus the physical barrier.
2. Play as part of an inclusive society.
3. The role of the professional.

**Table 3:** Barriers regarding participation in the playground for children with PD

Emotional barrier vs. Physical barrier	General inclusion	Role of professional
The social-emotional barrier is larger than the physical barrier. (pro)	The child has no friends in the neighborhood. (pro,par)	Parents do not know the importance of (independent) play. (par)
Parents help too much and think for the child, causing the child not to try and experience for itself. (pro, par)	Difficult to determine the number of children with disabilities in the neighborhood. (pro)	One-off events do not encourage persistence at playing in the playground. (pro)
Parents quit going to the playground due to negative experiences, such as being stared at by others. (pro)	Insufficient support for children with a disability in inclusive education. (pro, par)	Parents do not know the capabilities of their child and are not acquainted with how to play with their child. (pro, par)
Parents find it difficult to delegate. (pro,par)	Inclusion is difficult to organize. (pro)	Parents do not realize that their child can enjoy playing outside. (pro, par)
Parents have the feeling that there is no room for play without practicing motor tasks. They don’t want to feel as if they are practicing all the time. (par)	Parents of children without disabilities don’t want their children to play with a child with disabilities. They find the responsibility too great. (par)	Difficult to give a specific professional the role of improving play: this is highly dependent on the moment and involvement with the patient/client. (pro)
Parents need to work hard caring for their child and feel that they don’t have the time. (pro, par)	The level of play of children without disabilities at a young age is already very high, especially physically. (pro)	Professionals, mainly doctors, focus too much on health instead of participation. (par)
Growing into increasing deficit. (pro, par)	Playgrounds are not designed for all age ranges.(par)	Professionals are more focused on sport instead of play. (pro, par)
Child is scared and insecure, caused by over-protection by the parents. (pro)	Children without disabilities do not play outside often, so there are fewer children to play with outside. (pro, par)	The therapist uses play as a method, instead of a goal. (pro, par)
The surroundings of the playground are not safe. (par)	Not going to school in their neighborhood. (pro, par)	Professionals lack the competence to manage inclusion and stimulate play. (pro)
Some children stay dependent on help from someone else (cognitive, physical, social, emotional). (pro,par)	Children with disabilities play better together but there are few of them in one neighborhood. (pro)	The transition from therapy to participation is difficult due to the emotional barriers of parents and child. (pro)
The child compares itself with other children and feels ashamed. (par)	Playgrounds are physically inaccessible. (pro, par)	Therapy is directed towards the level of activities, not participation (in ICF-CY). (pro)
Difficulty with participating in play (cognitive, physical, social, emotional). (pro,par)	People tend to help and think too much for the children with disabilities, not allowing the child to experience for itself. (pro,par)	Therapy on location is difficult to implement due to a lack of time and money. (pro, par)
Playing inside and alone is preferred or feels safer, like playing with technology. (pro, par)	Children with disabilities do not know how to play with other children, with or without disabilities. (pro, par)	What a child needs is different for every child and remains personalized. (pro)
There are not enough opportunities to discover and experience the capabilities of the child in outside play, due to high emotional and physical barriers. (pro)	Children with disabilities are not included in society, causing inadequate acceptance and bad behaviour (e.g. bullying) towards them. (pro, par)	Professionals do not collaborate. (pro, par)
Child does not know its own capabilities. (pro)		
Parents of children with disabilities do not share experiences. (pro)		
Some children do not have an intrinsic motivation to explore in play. (pro, par)		
Wheelchair dependence. (pro)		
Extensive medical problems. (par, pro)		
Child accumulates negative experiences, like always losing, being unable to play, and bullying, causing the child to not enjoy play in the playground. (pro,par)		
In the first years of the child’s life, parents are emotionally not open to the idea of outside play due to mourning, processing, lots of worries, limited time, and diagnostics. (pro, par)		

*Pro* Professionals, *Par* Parents

**Table 4:** Facilitators regarding participation in the playground for children with PD

Emotional barrier vs. Physical barrier	General inclusion	Role of professional
Parents stimulate the child into independence at an early age. (par)	General inclusion, inclusive education, and inclusive daycare. (pro, par)	Pediatricians write prescriptions for therapy at home. (pro)
The child enjoys playing outside and playing together. (par)	A playground is a cozy place with friends. (pro, par)	Therapy directed towards participation level and at a functional location.(pro, par)
Parents stimulate the child to stand up for him or herself and be able to cope with negative reactions. (par)	Wheelchairs for other children to play with. (par)	Therapists who focus on play more and want to find out what motivates a child in play. (par)
Introduction of the playground when the child is young (+/− two years). (pro)	Child without disabilities is willing to help a child with a disability. (par)	
Known children, like brothers and sisters, that stimulate outside play. (pro, par)	Children who grow up with children with disabilities have a better perspective on children with disabilities. (pro, par)	
An ambulatory companion who facilitates play. (pro, par)	Young children are still flexible, therefore more easily familiarized with children with disabilities. (pro)	
Parents do know the importance of (independent) play (par)	Children know how to play together. (pro,par)	
Parents know the capabilities of their child. (pro, par)	Teacher stimulates inclusive play at school. (par)	
Parents of children with disabilities inspire or inform each other to play outside. (pro, par)	No competitive play. (pro)	
Child knows its own capabilities. (pro)	Fun play equipment: nest or wheelchair swing or carousel; small field with goals, hills, tunnels, and bridges; interactive elements with sand and water; a fort, ship, or house to play in or under; room for fantasy game; trail for wheelchairs but also bicycles; and steps, etc. (pro, par)	
A positive experience in the playground. (pro)		
Good wheelchair skills. (pro, par)		
Good social skills. (pro, par)		
The child has friends in the neighborhood. (pro)		
There is another child with a disability that plays outside (role model). (par)		
Parents equip their garden as a playground, which attracts other children to come and play. (par)		
Involved neighborhood association.(pro, par)		
Good wheelchair for activities. (pro)		
Indoor playgrounds are much fun. (pro, par)		

*Pro* Professionals, *Par* Parents

**Table 5:** Solutions regarding participation in the playground for children with PD

Emotional barrier vs. Physical barrier	General inclusion	Role of professional
Parents need to be coached and empowered to help them overcome social/emotional barriers. (pro, par)	More education to abolish the stigma of children with disabilities in society.(pro)	Professionals need to educate parents and close associates about the importance of play. (pro, par)
Role models, like paralympic athletes, can inspire the child. (pro)	All playgrounds need to be adapted for physical accessibility. (pro)	Involve parents in the therapy. (pro)
Support parents in letting go and allowing children to try and experience for themselves. (pro, par)	Increased general inclusion, inclusive education, and inclusive daycare. (pro, par)	Interventions need to be long-term before the behavioral change takes place. (pro)
Introduce a playmate, within their network or from a volunteer project. Parents and/or child go together with another family, another child without disabilities, or an ambulatory companion. (pro)	Increased general acceptance of children with a disability. (pro)	Therapy at the functional location. (pro)
Safe environment with regards to traffic, equipment, and shelter. (pro, par)	Health professionals and teachers need to be made competent in play and inclusion. (pro, par)	Intervention playing outside needs to start around the age of 2 years. (pro)
Parents need to be made aware that participation in play is not a physical problem but mostly a social problem. (par)	A therapist could make parents competent in stimulating inclusive play. (par)	Cluster information about inclusive play, for example in an app. (pro)
Organize so that parents of children with disabilities meet often, share experiences about play, and inspire each other. (pro)	Increased support for children in inclusive education, like ambulatory companions. (pro)	Support parents to take responsibility. (pro)
Teach the child his or her own capabilities and boundaries and increase self-esteem. (pro)	The playground should be a challenging environment. (pro, par)	All professionals have a role in stimulating play and inclusion. (pro)
Show parents what the possibilities are by involving them in therapy and during play weekends, play courses/workshops. (pro, par)	Structurally organized activities, like inclusive playground sports. (pro, par)	Professionals need to be all-round. (pro)
Support and coach parents with mourning and acceptance. (pro)	The network around the parents and child needs to be involved and needs to know how to play with and support the child during outside play. (pro)	Professionals need to be made aware of their role in stimulating play and inclusion. (pro)
Reduce care burden. (pro)	Playground close to home. (pro, par)	Increase cooperation in multidisciplinary teams. (pro, par)
Children enroll with a group from special education for a play activity, therefore not being the only child with disabilities. (pro)	Behavioral change towards children with disabilities encouraged by advertisements and education that reach the whole society. (pro, par)	Education takes place during regular visits to the professional and during play weekends, play courses/workshops. (pro)
Wheelchair skills training. (pro)	Wheelchairs for other children to play with. (par)	Professionals can suggest play as a therapy goal. (pro)
Social skills training. (pro, par)	Parents give education in the neighborhood about their child. (pro)	Professionals should focus more in therapy on participation at home, at school, or in the neighborhood. (pro, par)
Help parents to find a way of playing that stimulates the intrinsic motivation of the child. (pro)	Courses about inclusive play in education, for children and teachers. (pro, par)	

*Pro* Professionals, *Par* Parents

### Emotional barrier vs. physical barrier

In order to facilitate active and inclusive outdoor play for children with PD, the environment needs to be physically accessible: *“… naturally, when you have a playground, you strive to have accessibility …, so a child with a disability feels that he is being thought of. That is already a first thing …”* [professional 1]. However, all parents and professionals say improving physical accessibility alone will not lead to increased participation, since emotional barriers also prevent children from participating in playgrounds.

Most parents state they were not able to even think of play as an option in the first years of their child’s life since they were still preoccupied with mourning, processing, and everyday struggles. Professionals share the same experiences: *“I think at first, when they are so young, they want their child to sit and walk. There are so many basic questions, that they only start thinking about play later on”* [professional 10]*.* Children and parents need to realize that the child can enjoy him- or herself in the playground. However, parents also need to realize they cannot always prevent the child from having a negative experience. All professionals state that parents find it difficult to let go of their child and worry about her or his safety when the child is able to or is allowed to play with other children. *“You often see that the child is able to do it physically, but that it is just really the connection between the child and parents and being afraid that it is socially incapable, rather than physically incapable, of getting there. …cannot keep up with the other children … also cannot handle conflicts. I think that is what parents encounter”* [professional 13]*.* Most parents and professionals believe overprotection has a negative effect on the self-esteem, autonomy, and the self-solving ability of the child: *“… the key is actually, that they do everything for the child, so the child doesn’t develop self-esteem, that he/she is able to do something …”* [professional 1]*.* The lack of self-esteem, autonomy and self-solving abilities are the building blocks of the emotional barrier.

Many professionals observe the presence of friends as an important factor: *“… I think they really don’t care … if there is either a swing, a seesaw, or a jungle gym. It is more important they are with children from the neighborhood …”* [professional 3]*.* A majority of parents describe difficulties their child has connecting with typically developing children. A parent describes the example of her 10-year-old daughter: *“… that she now tries to connect with the four-year-olds, but she will not ask: can I play along? She just waits … until she gets an offer, or that she can secretly take a scoop (of sand) and that the* [other] *child actually finds it okay”* [parent 4]*.* On top of that, a child often grows into deficit, meaning the difficulties experienced with, for example, low cognition increases with the years, when compared to typically developing peers. Growing into deficit is an important negative factor in making friends. According to several parents, a possible way to decrease the social barrier might be for the child to go to the playground with a peer without a disability. *“It makes it easier for other children to make contact when the child with a disability is accompanied by a child they already know and it is easier for the child with a disability to feel equal”* [parent 6]. However, some children with PD need physical help and it is unclear whether another child could provide enough assistance. Moreover, physical problems also play a role in engaging with peers. Usually, typically developing children are faster, stronger, and more skilled. A parent reflects on this problem: *“She wants to play together, but the pace of other children is much higher … That’s why she sometimes doesn’t mind to just play alone in the garden … other children go outside and she goes her own way”* [parent 10].

On top of these barriers, some parents state they are sometimes tired of stimulating their child in play and they feel everything turns into a therapy session. Taking care of children with PD is emotionally demanding for parents, which leaves little or no emotional reserves for recreative trips to the playground. In order to decrease these barriers, an initiative has been suggested where families would be paired when going to the playground.

### Play as part of an inclusive society

All parents and professionals believe behavioral change is needed in the whole society towards the inclusion of children with PD. “*I do not think it is possible to say that by tomorrow we make the playground this way, we take into account these conditions, we support the parents and 1.5 years later or 2 years later all children with disabilities play in a playground…I believe it takes at least 10 years for this to become normal. This is behavioral change in my opinion, within families of children with disabilities…but also within society to make everything accessible”* [professional 1]*.* Behavioral change starts with raising awareness about play and inclusion within society, for example by running large-scale campaigns focusing on the current way society sees children with PD. Several parents say that typically developing children perceive children with PD as unusual and children with PD were sometimes bullied because of this. As a result, children with PD encounter difficulties in connecting with peers. One parent reported an example where their child was not allowed to play with typically developing children because parents of these children sometimes felt the responsibility of taking care of children with a disability was too great. Many parents and professionals say that children with PD are stigmatized. They hope, by changing the stigma, society will be able to truly include children with PD in everyday life in the neighborhood. All participating parents and professionals found it important to familiarize typically developing children and their parents with children with PD by meeting each other*: “… but he has little connection with the kids from nearby. Most children only see the wheelchair and then they ask me, what does he have? Is something wrong with his legs? Then I think, you can also ask him. Yes, I find that very difficult”* [parent 7]*.* Encouraging young children with PD to play in playgrounds is not only important for the child with a disability but also for children without PD and adults. Most parents and professionals feel that, when children without PD grow up with a child with a disability, they do not see the disability but instead they see the child. *“There is a huge gap in society, which can be very annoying at an older age … That’s just how society works. And then the government pays lip service to a society where participation plays a central role. But if you don’t grow up together, you don’t know each other”* [parent 7]*.* One example parents and professionals mention that preserves this gap in society and holds children with PD back from making friends in the neighborhood is special (i.e. regional not local) education. Both parents and professionals say that inclusive education and inclusive daycare could improve the integration of children with PD in society: *“Special education is, what I just said, a regional school, so friends are in school and do not live around the corner…I now have a number of children who changed from special education to regular local education once a week…and those parents all indicated that there is more participation in play in the neighborhood”* [professional 3]*.*

### Role of professional

A few parents are critical of the professionals involved in their child’s development, especially when these professionals do not consider play to be important. Parents say, for example, some doctors focus solely on medical issues and often do not consider the broader perspective. Many therapists use play as a method of achieving therapy goals but rarely set therapy goals focused on play: *“Yes, I apply it in my therapy, when children have to learn something, then I use play as a method, which I do playfully. But how many times was I really focused on whether a child could play? Well, then that is pretty limited”* [professional 18]*.* Some professionals state that the reason they are not focused on play and inclusion of children with PD is a lack of awareness about their role and the importance of play, and the fact they lack skills. However, therapists find it very important to provide parents and children with enough tools to ensure play is part of the child’s everyday life. Parents and professionals state that providing therapy on the level of participation in real-world situations is crucial but is not at present embedded in regular therapy: *“Yes … we were always here in the practice* [setting] *… But I’d rather that the therapist tells me there (in the playground) … what I can do with my daughter, or that what I do is good”* [parent 5]*.* Some professionals feel fostering play as part of the everyday life of children with PD should be the aim of long-term therapy. However, therapists also experience a financial barrier when treating children with PD in the home environment for a longer period of time. This financial barrier is caused by the reluctance of physicians in prescribing home-treatment referrals and the amount of time and, therefore money, these home treatments require.

In order to make parents aware of the importance of play, professionals should stimulate, coach, and empower parents: *“… create awareness with parents. I think that this process should start at a very early age* “[professional 18]*.* Some parents are not always aware that play is important for the development of their child. Children need to be encouraged to play in playgrounds at a young age, to let play become a normal part of their lives: *“Children will enjoy it more, I think, when you start at an early age, it becomes natural. I think the longer you wait before you go, the less they* [children with PD] *are going to enjoy it for themselves”* [professional 19]*.* Moreover, when a child is young, it is normal to provide assistance and parents can adapt to gradually let go of their child. Many professionals stated a solution might be to support parents by integrating play into regular interventions, including groups, and organize play weekends or play workshops.

## Discussion

The purpose of this study was to describe parents’ and professionals’ perspectives on barriers, facilitators and solutions influencing participation of children with PD in physically active play in playgrounds in the Netherlands. The results indicate that participation in playgrounds for children with PD is complex and influenced by multiple personal and environmental factors. Besides physical barriers, the emotional barriers seem to be the most important factors holding children with PD and their parents back from active participation in the playground. There were no fundamental differences observed in the perspectives of parents and professionals.

### Emotional barrier vs. physical barrier

Results of our present study correspond with previous research describing children with disabilities facing exclusion in a playground, because of physical inaccessibility [23–28]. However, since the emotional barrier seems crucial, it is interesting to notice literature about children with PD playing outside is mainly focused on physical aspects [23–28]. A study that did focus on both the physical and the emotional barriers in relation to play for children with disabilities found key themes similar to the findings of our present study, such as social exclusion by peers, lack of friends, not being able to adjust the type of outdoor play, the need for an adult to facilitate play, and the attitude of professionals to, and parents’ worries about the risks of outdoor play [28]. They also underline the necessity to focus on both the physical and the emotional barriers to increase participation of children with PD [28]. So besides improving accessibility of playgrounds, developing additional interventions focusing on experienced emotional barriers seem essential. Two studies on participation and happiness of parents and children with disabilities state that in order to increase participation of children with disabilities policy’s and interventions need to address factors (e.g. reducing stress associated with caring for a child with a disabilities, improving social skills of the child) on a system-wide level such as community-level support groups, stress relief strategies and exercise [41, 42].

### General inclusion

One specific example of a system wide change is inclusive education and daycare. Separate, and thereby exclusive, education in the Netherlands (special versus regular) seems to increase problems with general inclusion. Evidence shows that 85% of children with disabilities attending special education in the Netherlands have few or no friends in their neighborhood [43]. Properly organized inclusive education and daycare decreases social emotional barriers and stimulates integration in the community [44–46].

### Role of professional

Both parents and professionals in this study expressed that professionals could help to overcome multiple barriers, especially emotional ones. Solutions mentioned were: home-based therapy in the playground, coaching and empowering parents to overcome social/emotional barriers, early-age intervention focusing on outside play starting around the age of 2 years, teaching children their own capabilities and boundaries and increasing self-esteem. These elements coincide with implications Palisano et al. (2012) drew in their study regarding participation-based therapy for children with PD [47]. Furthermore, coaching and cooperating with parents are important elements of Family-Centered Care, and are known to support children and parents applying learned skills in natural environments [48]. Moreover, coaching is considered to increase knowledge, self-solving abilities, and self-advocacy skills of both children and parents and can help them develop strategies to overcome barriers leading to increased participation in the playground [48–50].

The use of behavioral change interventions in pediatric rehabilitation aimed at the interaction of children with disabilities and their parents and the physical and social context seems promising [51–54]. However, nowadays, professionals such as pediatric physiotherapist and occupational therapist still seem too much focused on function and activity, f.e. motor skills such as learning to walk or cycle, rather than on changing or involving the environment and thus participation. Professionals still seem to be insufficiently aware of existing social barriers, while the social aspect is essential in inclusive outdoor play [51, 55–58].

Professionals in this study reported barriers in the organization of care for children with PD in the Netherlands. One example is the difficulty of providing home-based therapy in the natural environment, as children with PD in the Netherlands typically receive their therapy at their regional special education school or within private practices. Since these schools are not located in the neighborhood of children with disabilities, it is very time- and cost consuming for the therapist to provide therapy in children’s living situations.

### Strengths and limitations

Several strengths and limitations were present in our study. The method of this study was described using the Consolidated criteria for reporting qualitative studies (COREQ) [36]. We conducted the analysis with two independent researchers, included critical peer review and member checking which enhanced the credibility, conformability, and reliability of this qualitative study [35, 37]. Professionals with a diverse range of experiences participated in our study, leading to a broad overview of perspectives about play in the playground for children with disabilities. The selection of participating professionals positively affected the heterogeneity of the research group. In terms of medical diagnosis, gender, age, and mobility of the child, heterogeneity of parents and professionals was particularly high. To our knowledge, the present study is the first in the Netherlands to analyze participation in physically active play in the playground for children with PD.

A limitation of our study was that parents were recruited at events for children with PD and approached through ‘The Playground Gang’ (De Speeltuinbende). There is a chance parents who agreed to participate in our study were those who already found play important for their child. Confirmation bias may have been present since the interviews were conducted by only one interviewer. We tried to prevent bias by using an interview guide. In addition, only two of the five focus group meetings consisted of six to ten participants, the ideal range [35], which could have influenced the results: however, interactions in focus group meetings seemed adequate.

### Implications for the future

As children are the main stakeholders in this project, future research to find additional facilitators, barriers and solutions should include their perspectives. Furthermore, a system wide intervention could be developed based on the outcomes of this study. This intervention should focus on and be created in co-creation with health care professionals, policy-makers, the government, and other stakeholders. These solutions should in turn be evaluated with children (with and without PD), parents, and professionals, investigating the feasibility of these interventions to increase play in playgrounds for children with PD in the long term.

## Conclusion

According to parents and professionals, the main reason for children with PD not participating in play in the playground is the emotional barrier that both parents and their children experience. Professionals and parents see many routes for improvement, mainly with regard to the organization of services for children with PD. Suggested solutions all focus on empowering and coaching both children and parents. To enable participation for children with PD, change is needed in all layers of society.

## Supplementary Information

**Additional file 1.** Consolidated criteria for reporting qualitative studies (COREQ): 32-item checklist.

**Additional file 2.** Topic list: parents.

**Additional file 3.** Topic list: professionals.

**Additional file 4.** Questionnaire general information parents.

**Additional file 5.** Questionnaire general information professional.

## Data Availability

The datasets used and analysed during the current study are available from the corresponding author on reasonable request.

## Abbreviation

PD: Physical disabilities
