# Supplementary material for: Barriers, facilitators and solutions for active inclusive play for children with a physical disability in the Netherlands: a qualitative study
Source: BMC Pediatr. 2021 Aug 28;21:369. doi: 10.1186/s12887-021-02827-5 (PMC8401178; doi:10.1186/s12887-021-02827-5)
Supplement: Supplementary file 1 — Additional file 1. Consolidated criteria for reporting qualitative studies (COREQ): 32-item checklist. [file 12887_2021_2827_MOESM1_ESM.docx]

**Appendix 1** Consolidated criteria for reporting qualitative studies (COREQ): 32-item checklist

BARRIERS, FACILITATORS AND SOLUTIONS FOR ACTIVE INCLUSIVE PLAY FOR CHILDREN WITH A PHYSICAL DISABILITY IN THE NETHERLANDS: A QUALITATIVE STUDY.

van Engelen L,^1,2^ Ebbers M,^1,2^ Boonzaaijer M,^1,2^ Bolster EAM,^1,2^ van der Put EAH^3^, Bloemen MAT*^1,2^

^1^HU University of Applied Sciences Utrecht, Institute of Human Movement Studies, Master Pediatric Physiotherapy, Utrecht, the Netherlands, ^2^HU University of Applied Sciences Utrecht, Research Group Lifestyle and Health, Research Centre for Healthy and Sustainable Living, Utrecht, the Netherlands, ^3^De Speeltuinbende, Amsterdam, the Netherlands

*manon.bloemen@hu.nl

**Consolidated criteria for reporting qualitative studies (COREQ): 32-item checklist**

We copied and pasted literal text from the manuscript so to information is easily traceble.

Domain 1: Research team and reflexivity Personal Characteristics

1. *Interviewer/facilitator. Which author/s conducted the interview or focus group?
   The interviews were led by ME (MSc. pediatric physical therapist (PPT)). Focus group meetings were led by LvE (MSc. PPT).*
2. *Credentials. What were the researcher’s credentials? E.g. PhD, MD. ME (MSc. pediatric physical therapist, (PPT)). LvE (MSc. PPT).*
3. *Occupation. What was their occupation at the time of the study? ME ( MSc. pediatric physical therapist (PPT)). LvE (MSc. PPT).*
4. Gender. Was the researcher male or female? *Irrelevant*
5. Experience and training. What experience or training did the researcher have? *The research team, consisting of one experienced (> 15 years) MSc. pediatric physical therapist/pedagogue (MBO), two experienced (> 10 and 15 years respectively) PhD. pediatric physical therapists (EB, MB) and two junior researchers (<5 years) with MSc. pediatric physical therapy background (ME and LVE). The research team was trained for qualitative research.*

Relationship with participants

1. Relationship established. Was a relationship established prior to study commencement? *Participants were recruited through different routes, including our network, treatment practices found on the internet, and by networking at a wheelchair event.*
2. Participant knowledge of the interviewer. What did the participants know about the researcher? e.g. personal goals, reasons for doing the research. *All participants were provided with a standardized information letter. Prior to interviews, focus group meetings and member check group meetings, every participant gave written informed consent.*
3. Interviewer characteristics. What characteristics were reported about the interviewer/facilitator? e.g. Bias, assumptions, reasons, and interests in the research topic. *Focus group meetings were led by one moderator LvE (MSc. PPT).
   The interviews were solely led by the moderator ME (MSc. PPT).
   two junior researchers (<5 years) with a MSc. pediatric physical therapy background (ME and LVE).*

Domain 2: Study design Theoretical framework

9. Methodological orientation and Theory. What methodological orientation was stated to underpin the study? e.g. grounded theory, discourse analysis, ethnography, phenomenology, content analysis. *This case study employed a qualitative descriptive design, with a thematic analysis (33) and has a basis in social constructivism (34,35). In this approach, questions remain broad to encourage participants to construct their meaning, supported by the interaction with others (the social part of social constructivism) (34,35).*

Participant selection
10. Sampling. How were participants selected? e.g. purposive, convenience, consecutive, snowball *We used purposive sampling to assure variation in GMFCS score, age, background, family composition, and environment of the parents that we interviewed. For the professionals, we included participants with different levels of experience and different professions(39). Maximum variation of demographic origin was aimed for. Participants were recruited by flyers through different routes, including our network, treatment practices found on the internet, and by networking at a wheelchair event. In order to reduce the burden of travel and time, the focus groups were formed by convenience, rather than variation by age, gender, profession, or level of experience.*

11. Method of approach. How were participants approached? e.g. face-to-face, telephone, mail, email. *Participants were recruited by flyers through different routes, including our network, treatment practices found on the internet, and by networking at a wheelchair event.*

12. Sample size. How many participants were in the study? *From November 2017 to February 2018, five focus group meetings with professionals (n = 25) and 12 semi-structured interviews with parents were conducted.
In addition, no new information was discussed by the participants (professionals n = 15; parents n = 9) during the member checks.*

13. Non-participation. How many people refused to participate or dropped out? Reasons? *There were no drop-outs.*

14. Setting of data collection. Where was the data collected? e.g. home, clinic, workplace *Both the individual interviews and the focus group interviews were conducted at various locations such as a rehabilitation center, the HU University of Applied Sciences Utrecht, pediatric physical therapy practice, and at the participant’s home.*

15. Presence of non-participants. Was anyone else present besides the participants and researchers? *Focus group meetings were led by one moderator LvE (MSc. PPT). There was one observer who made fieldnotes and supported the moderator in making sure all topics of the topic list were discussed.
The interviews were solely led by the moderator ME (MSc. PPT) who made field notes to make sure all the topics were discussed and to register particularities and non-verbal reactions.*

16. Description of sample. What are the important characteristics of the sample? e.g. demographic data, date. *We included parents of children with PD comparable to a score of I-IV on the GMFCS and aged 2-12 years old and professionals who were experienced in working with children with PD in the field of physical activity. The exclusion criterium was insufficient in the Dutch language.
For specific details about the participants see table 1 and 2*

Data collection
17. Interview guide. Were questions, prompts, guides provided by the authors? Was it pilot tested? *The parents and professionals were interviewed separately with comparable topic lists, so any differences and similarities between these groups could become evident. However, during the face of date collecting the topic lists will be adjusted for usability reasons and by gained knowledge regarding the subject. The topic lists were created by the research team, consisting of one experienced (> 15 years) MSc. pediatric physical therapist/pedagogue (MBO), two experienced (> 10 and 15 years respectively) PhD. pediatric physical therapists (EB, MB) and two junior researchers (<5 years) with a MSc. pediatric physical therapy background (ME and LVE). The research team was trained for qualitative research.*

*In all interviews, the moderator posed questions to which the participants responded. The participants were not directed towards any particular preconceived response.*

18. Repeat interviews. Were repeat interviews carried out? If yes, how many? No, irrelevant to mention in the manuscript.

19. Audio/visual recording. Did the research use audio or visual recording to collect the data? *All interviews were filmed and audio-taped.*

20. Field notes. Were field notes made during and/or after the interview or focus group? *The interviews were solely led by the moderator ME (MSc. PPT) who made field notes to make sure all the topics were discussed and to register particularities and non-verbal reactions.
Focus group meetings were led by one moderator LvE (MSc. PPT). There was one observer who made fieldnotes and supported the moderator in making sure all topics of the topic list were discussed.*

21. Duration. What was the duration of the interviews or focus group? *The average duration of the meetings was 90 minutes and the interviews with parents 46 minutes.*

22. Data saturation. Was data saturation discussed? *To assess the likelihood of saturation (38,39), three member checks with new participants were performed after the data collection was finished. One member check group consisted of parents, the other two of professionals. When no new information arose during the member check meetings, saturation was assumed.*

23. Transcripts returned. Were transcripts returned to participants for comment and/or correction? *No, the transcripts were not returned, but there were member check meetings.*

Domain 3: analysis and findings Data analysis

24. Number of data coders. How many data coders coded the data? *The analyses were conducted independently by two trained researchers (ME and LvE) and then compared. In the case of disagreement, a third researcher (MB) was consulted to achieve consensus. Then the generated codes were searched for similarities and, when possible, merged. From the resulting codes, several overarching themes were identified and thoroughly discussed within the research team. To prevent research bias, critical peer debriefing occurred after each step. (38,39).*

25. Description of the coding tree. Did authors provide a description of the coding tree? *The first step was familiarization with the data by reading the transcripts several times. The second step was identifying fragments and coding these fragments to distinguish between barriers, facilitators, and solutions. Barriers and facilitators were defined as aspects already present and which have a negative or positive influence on physically active participation by children with PD in playgrounds. Solutions were defined as factors, not yet present but with a potentially positive influence on participation in playgrounds. During the third step, searching for a broader lever of themes within the codes. The fourth step specified the detailed reviewing of the theme in relation to the individual codes and to the entire data set to conclude if the themes display all the collected information. The fifth step was defining and naming the themes and distinguish themes from subthemes.*

26. Derivation of themes. Were themes identified in advance or derived from the data? *A thematic analysis was performed using an inductive strategy (33)*

27. Software. What software, if applicable, was used to manage the data? *A thematic analysis was performed using an inductive strategy (33) with MAXQDA2018 (version 18.0.4).*

28. *Participant checking. Did participants provide feedback on the findings? To assess the likelihood of saturation (38,39), three member checks with new participants were performed after the data collection was finished. One member check group consisted of parents, the other two of professionals.*

Reporting

29. Quotations presented. Were participant quotations presented to illustrate the themes/findings? Was each quotation identified? e.g. participant number. *The most important issues are discussed in the text and supported with relevant quotes.*

30. Data and findings consistent. Was there consistency between the data presented and the findings? *Yes.*

31. Clarity of major themes. Were major themes clearly presented in the findings? *Yes.*

32. Clarity of minor themes. Is there a description of diverse cases or discussion of minor themes? *Yes.*
