# Supplementary material for: Barriers, facilitators and solutions for active inclusive play for children with a physical disability in the Netherlands: a qualitative study
Source: BMC Pediatr. 2021 Aug 28;21:369. doi: 10.1186/s12887-021-02827-5 (PMC8401178; doi:10.1186/s12887-021-02827-5)
Supplement: Supplementary file 2 — Additional file 2. Topic list: parents. [file 12887_2021_2827_MOESM2_ESM.docx]

**Appendix 2 Topic list: parents**

BARRIERS, FACILITATORS AND SOLUTIONS FOR ACTIVE INCLUSIVE PLAY FOR CHILDREN WITH A PHYSICAL DISABILITY IN THE NETHERLANDS: A QUALITATIVE STUDY.

van Engelen L,^1,2^ Ebbers M,^1,2^ Boonzaaijer M,^1,2^ Bolster EAM,^1,2^ van der Put EAH^3^, Bloemen MAT*^1,2^

^1^HU University of Applied Sciences Utrecht, Institute of Human Movement Studies, Master Pediatric Physiotherapy, Utrecht, the Netherlands, ^2^HU University of Applied Sciences Utrecht, Research Group Lifestyle and Health, Research Centre for Healthy and Sustainable Living, Utrecht, the Netherlands, ^3^De Speeltuinbende, Amsterdam, the Netherlands

*manon.bloemen@hu.nl

**Objective:**

The objective is to analyze facilitators, barriers and solutions that are important for active and inclusive outdoor play for children with physical disabilities (aged 2-12 years) from the parents perspective.

**Definitions**

Play was defined as: *“any behavior, activity or process initiated, controlled and structured by children themselves’. Caregivers may contribute to the creation of environments in which play takes place, but play itself is non-compulsory, driven by intrinsic motivation and undertaken for its own sake, rather than as a means to an end.”* (1)

A playground was defined as: all possible exterior places where play can originate.

**Themes**

- Current situation of playing outside

- Technical aspects

- Physical aspects

- Cognitive aspects

- Behavioral aspects

- Parents

- School environment

- Quality of collaboration between parents and health care professionals in relation to active inclusive play.

**Interview questions and topic list:**

**Current situation of playing outside**

Main question: Does your child often play outside in playgrounds and what are your experiences with this?
- Play where? (Might it also be an indoor playground?) How? How often?
- If your child does not play in the playground, why not?
- Play how? Physically active or not? (explain definition of physical activity)
- Play how? Together or alone?
- Play how? Independent or with help/guidance?
- What do you find important as a parent as regards inclusive active playing in playgrounds?
- Why is this important to you?
- Views on playing outside and playing with peers; interest and risk?
- Views on play and physical activity; benefits and risks?

**Technical aspects of playground**

Main question: What is important in a playground in order to make playing with peers possible and attractive to your child?

- Playground equipment; its form, what facilitates play and what is a barrier? Can you explain?

- Accessibility

- For wheelchair-driving children; the possibility of getting out of the wheelchair or making transfers.

- Crowded or quiet?

- What would you like for your child that is currently not present in the playground in your neighborhood, and why?

**Physical aspects**

Main question: Do your child's physical abilities play a role in whether or not he/she plays in playgrounds in a physically active way, and why?

- What physical aspects are barriers?

- Is your child driving a wheelchair?

- Is help necessary while playing?

**Cognitive aspects**

Main question: Are there cognitive factors in your child's behavior that can affect inclusive play? If so, can you explain?

- Why do these factors play a role?

- Problems with processing stimulus, difficulty with crowds, etc.

- Communication (with other children), speech-language problems

- Are there factors in a playground that can positively or negatively affect cognitive problems? Can you explain your answer?

**Behavior**

Main question: Are there factors in your child's behavior that influence playing with peers and why?

- How do friends interact with your child in the playground? And vice versa?

- How do unknown children interact with your child in the playground? And vice versa?

- Influence of self-confidence and self-image

**Parents (role)**

Main question: What do you do to stimulate your child to pursue physically active joint play in playgrounds?

- Do you go and play together with your child in the playground? Explain your answer

- What is your role in stimulating your child in physically active joint play (e.g. only helping, playing together, directing)?

- How much help do you offer

- Why that amount of help?

- Siblings?

- What would you like to do better yourself and how could you be helped?

**School environment**

Main question: How do you think school deals with active joint play in relation to your child?

- Why are you satisfied or not about school and play?

- Do you have an impression of how your child plays in the schoolyard?

- How is your child guided in this?

- Is playing together at school different than at home?

- What could be better?

**Experiences of parents with health care professionals**

Main question: How do you think health care professionals involved with your child deal with active joint play?

- Are health care professionals involved? (explain the concept of health care professionals)

- What kind of health care professionals?

- Do the social workers play a role in relation to playing outside? If so, how?

- What do you do with instructions from the health care professionals?

- What could health care professionals do better?

- Do you think the therapist(s) know the playgrounds in the area?

- Does the therapist come to your home? Do you see an advantage for treatment in your own environment and why?

- How do you feel about the attention paid to the topic active inclusive play by foundations?

- How do you feel about the societies look in active inclusive play?
