# Supplementary material for: Barriers, facilitators and solutions for active inclusive play for children with a physical disability in the Netherlands: a qualitative study
Source: BMC Pediatr. 2021 Aug 28;21:369. doi: 10.1186/s12887-021-02827-5 (PMC8401178; doi:10.1186/s12887-021-02827-5)
Supplement: Supplementary file 3 — Additional file 3. Topic list: professionals. [file 12887_2021_2827_MOESM3_ESM.docx]

**Appendix 3 Topic list: professionals**

BARRIERS, FACILITATORS AND SOLUTIONS FOR ACTIVE INCLUSIVE PLAY FOR CHILDREN WITH A PHYSICAL DISABILITY IN THE NETHERLANDS: A QUALITATIVE STUDY.

van Engelen L,^1,2^ Ebbers M,^1,2^ Boonzaaijer M,^1,2^ Bolster EAM,^1,2^ van der Put EAH^3^, Bloemen MAT*^1,2^

^1^HU University of Applied Sciences Utrecht, Institute of Human Movement Studies, Master Pediatric Physiotherapy, Utrecht, the Netherlands, ^2^HU University of Applied Sciences Utrecht, Research Group Lifestyle and Health, Research Centre for Healthy and Sustainable Living, Utrecht, the Netherlands, ^3^De Speeltuinbende, Amsterdam, the Netherlands

*manon.bloemen@hu.nl

**Objective:**

The objective is to analyze facilitators, barriers and solutions that are important for active and inclusive outdoor play for children with physical disabilities (aged 2-12 years) from the professional’s perspective.

**Definitions**

Play was defined as: *“any behavior, activity or process initiated, controlled and structured by children themselves’. Caregivers may contribute to the creation of environments in which play takes place, but play itself is non-compulsory, driven by intrinsic motivation and undertaken for its own sake, rather than as a means to an end.”* (1)

A playground was defined as: all possible external places where play can originate.

**Environmental ('external') factors influencing levels of functioning**

**Physical Environment and Societal Structures**

- School
  - Inclusive education
  - Distance of school from home
- Government
- Professionals
- General inclusion
- Inclusive daycare
- Information
  - To whom
  - By whom
  - What sort
  - Ease of location
  - Availability

**The social environment**

*Different standard forms of playing*

- Playing tag
- Hide and seek
- Ball games
- Competitions
- Parkour
- Fantasy game

*Playing together with and without disabilities*

- Knowing abilities
- Creative
- Leadership

*Play environment*

- Accessibility
- Playmates
- Other children with disabilities
- Parents of children without disabilities
- Materials
- Challenging environment
- Risk/safety
- Supervision
- Sanitary facilities

**Personal ('internal') factors influencing levels of functioning**

*Child with disabilities*

- Assistance
- Examples
- Fear
- Social skills
- Physical skills
- Age
- Joy

*Parent of child with disabilities*

- Fear
- Mourning
- Providing assistance
- Thoughts about play
- Seeing possibilities
- Role of parents during play
- General burden of having a child with disabilities
