# Supplementary material for: Barriers, facilitators and solutions for active inclusive play for children with a physical disability in the Netherlands: a qualitative study
Source: BMC Pediatr. 2021 Aug 28;21:369. doi: 10.1186/s12887-021-02827-5 (PMC8401178; doi:10.1186/s12887-021-02827-5)
Supplement: Supplementary file 4 — Additional file 4. Questionnaire general information parents. [file 12887_2021_2827_MOESM4_ESM.docx]

# **Appendix 4 Questionnaire general information parents**

BARRIERS, FACILITATORS AND SOLUTIONS FOR ACTIVE INCLUSIVE PLAY FOR CHILDREN WITH A PHYSICAL DISABILITY IN THE NETHERLANDS: A QUALITATIVE STUDY.

van Engelen L,^1,2^ Ebbers M,^1,2^ Boonzaaijer M,^1,2^ Bolster EAM,^1,2^ van der Put EAH^3^, Bloemen MAT*^1,2^

^1^HU University of Applied Sciences Utrecht, Institute of Human Movement Studies, Master Pediatric Physiotherapy, Utrecht, the Netherlands, ^2^HU University of Applied Sciences Utrecht, Research Group Lifestyle and Health, Research Centre for Healthy and Sustainable Living, Utrecht, the Netherlands, ^3^De Speeltuinbende, Amsterdam, the Netherlands

*manon.bloemen@hu.nl

# This questionnaire consists:

# - 12 questions (page 2 and 3)

# Filling in the questionnaire will take about 10 minutes.

# Please return the completed questionnaire within a week via the enclosed reply envelope. If you have any questions, you can always contact Manon Bloemen [manon.bloemen@hu.nl](mailto:manon.bloemen@hu.nl)

# **Study number:**

# **General data family**

**You have a:**

single parent family

father

mother

two-parents family

father and mother

two fathers

two mothers

(if you have a family with two fathers / two mothers, please indicate in futher questions; father 1, father 2 / mother 1, mother 2).

**Gender of your child:**

boy

girl

**Medical diagnose of your child with a disability:**

……………………………………………………………………………..

**Date of birth mother:** ……./ …… / 19….

**Date of birth father:** ……./ …… / 19….

**Date of birth child with a disability:** ……./ …… / ..….

**Do you have multiple children?**

| Gender | Date of birth | Medical diagnose, yes/no |
| --- | --- | --- |
|  |  |  |
|  |  |  |
|  |  |  |
|  |  |  |
|  |  |  |

| **Country of origin.** | **child** | **father** | **mother** |
| --- | --- | --- | --- |
| The Netherlands |  |  |  |
| Turkey |  |  |  |
| Morocco |  |  |  |
| Suriname |  |  |  |
| Netherlands Antilles |  |  |  |
| Otherwise, namely: |  |  |  |

**Daily method of locomotion child?**

 always walking

 walking, using a walking aid (such as crutches, etc.)

 walking, wheelchair only for long distances

 walking and wheelchair driving and using a walking aid

 always by wheelchair

| **Education in the Netherlands:** | **father** | **mother** |
| --- | --- | --- |
| Low |  |  |
| Middle |  |  |
| High |  |  |

**Profession father:**……………………………………………………………….....

**Profession mother:**…………………………………………………………………

| **Worktime (hours/week)** | **father** | **mother** |
| --- | --- | --- |
| 0 hour |  |  |
| 1-8 hours |  |  |
| 9-16 hours |  |  |
| 17-32 hours |  |  |
| > 32 hours |  |  |
