# Supplementary material for: Barriers, facilitators and solutions for active inclusive play for children with a physical disability in the Netherlands: a qualitative study
Source: BMC Pediatr. 2021 Aug 28;21:369. doi: 10.1186/s12887-021-02827-5 (PMC8401178; doi:10.1186/s12887-021-02827-5)
Supplement: Supplementary file 5 — Additional file 5. Questionnaire general information professional. [file 12887_2021_2827_MOESM5_ESM.docx]

# **Appendix 5 Questionnaire general information professional**

BARRIERS, FACILITATORS AND SOLUTIONS FOR ACTIVE INCLUSIVE PLAY FOR CHILDREN WITH A PHYSICAL DISABILITY IN THE NETHERLANDS: A QUALITATIVE STUDY.

van Engelen L,^1,2^ Ebbers M,^1,2^ Boonzaaijer M,^1,2^ Bolster EAM,^1,2^ van der Put EAH^3^, Bloemen MAT*^1,2^

^1^HU University of Applied Sciences Utrecht, Institute of Human Movement Studies, Master Pediatric Physiotherapy, Utrecht, the Netherlands, ^2^HU University of Applied Sciences Utrecht, Research Group Lifestyle and Health, Research Centre for Healthy and Sustainable Living, Utrecht, the Netherlands, ^3^De Speeltuinbende, Amsterdam, the Netherlands

*manon.bloemen@hu.nl

# This questionnaire consists:

# - 5 questions (page 2 )

# Filling in the questionnaire will take less than 10 minutes.

# Please return the completed questionnaire within a week via the enclosed reply envelope. If you have any questions, you can always contact Manon Bloemen [manon.bloemen@hu.nl](mailto:manon.bloemen@hu.nl)

# **Study number:**

What is your gender?

_________________________________________________________________________

What is your land of origin?

_________________________________________________________________________

What is your profession?

_________________________________________________________________________

What is your field of practice?

_________________________________________________________________________

How many years of working experience do you have?

_________________________________________________________________________
